# Induction of Transcription Factor Early Growth Response Protein 1 during HSV-1 Infection Promotes Viral Replication in Corneal Cells

**DOI:** 10.9734/BMRJ/2013/4817

**Published:** 2013-09-03

**Authors:** S. C. Hsia, L. P. Graham, G. R. Bedadala, M. B. Balish, F. Chen, R. W. Figliozzi

**Affiliations:** 1 Pharmaceutical Sciences, School of Pharmacy, University of Maryland Eastern Shore, 1 College Backbone Road, Princess Anne, MD 21853, USA

**Keywords:** Egr-1, HSV-1, lytic infection, viral replication, corneal cells

## Abstract

**Aims:**

To understand the mechanisms of Early Growth Response Protein 1 (Egr-1) induction upon HSV-1 lytic infection and its roles in regulating viral gene expression and replication.

**Study Design:**

Rabbit corneal cell line SIRC and other cell lines were infected by HSV-1 to investigate the Egr-1 induction and its occupancy on the viral genome in different conditions. UV-inactivated HSV-1 and a recombinant virus over-expressing Egr-1 were generated to evaluate the regulatory effects on viral gene expression and replication during the infection.

**Methodology:**

Egr-1 induction triggered by viral infection was determined by Western Blot analyses and immune-fluorescent microscopy. Real-time RT-PCR and a novel Cignal^™^ Reporter Assay were used for quantitative measurement of Egr-1 expression. Chromatin Immuno-precipitation (ChIP) was performed to address the Egr-1 occupancy to the viral regulatory sequences and the influence on viral replication was assessed by plaque assays.

**Results:**

Our results indicated that Egr-1 expression requires viral gene expression since the UV-inactivated HSV-1 failed to produce Egr-1 protein. Blockade of viral replication did not block the Egr-1 protein synthesis, supporting the hypothesis that HSV-1 replication was not essential for Egr-1 production. Chromatin immune-precipitation (ChIP) and RT-PCR assays demonstrated that induced Egr-1 was able to interact with key regulatory elements near HSV-1 immediate-early (IE) genes and promote viral gene expression. Recombinant virus overexpressing Egr-1 revealed that Egr-1 enhanced the viral replication and the release of infectious virus.

**Conclusion:**

Together these results concluded that HSV-1 triggers the expression of an important host transcription factor Egr-1 via a unique mechanism and benefit the viral gene expression and replication.

## 1. INTRODUCTION

HSV-1 developed many strategies to infect a broad range of human cell types. During infections the virus decomposed host RNA and turned off protein synthesis efficiently[1]. As a result, cellular proteins are selectively destroyed or stabilized to carry out new task. In addition, certain host proteins were not present but rapidly produced after the entry of virus into the cells. Early Growth Response type-1 (Egr-1) is one of the proteins that belong to this group]2].

Egr-1 is a Zinc finger binding protein that has many different names such as Nerve Growth Factor-Induced gene A (NGFI-A), Zinc finger binding protein clone 286 (zif268), etc. Its sequence exhibits homology to the Drosophila Kr finger probe (Krox-24). Egr-1 is a member of the immediate early gene superfamily, whose presence is robust and transient in the cell shortly after the stimulation without de novo protein synthesis[3]. Egr-1 has been linked to many biological functions including cell growth, survival, and transformation, etc. The gene product codes for a transcription factor, preferentially binds to the GC-rich sequence 5′-GCGGGGGCG-3′ [4].

Egr-1 is activated by several pathways which include p38/MAPK[5]JNK[6], and MEK/ERK [7], etc. Its expression is found to be correlated to infection by several viruses[2]. These evidences suggest that Egr-1 may play a role in viral gene expression and replication. Previously we demonstrated that Egr-1 was induced upon HSV-1 infection in cell lines such as Vero as well as SIRC, a corneal cell line[2]. However, the mode of action and its role during viral replication are unknown. In this study we investigate the mechanisms controlling Egr-1 expression by viral infection and to understand its effects on viral life cycles.

## 2. MATERIALS AND METHODS

### 

#### Cells, viruses, reagent, cell culture and infections

Vero (African green monkey kidney fibroblast, Cat#: CCL-81), SIRC (Rabbit cornea, Cat#: CCL-60), and HEK293 (Cat#: CRL-1573) cells were purchase from ATCC (Manassas, VA). Vero and HEK293 are grown in DMEM supplemented with 10% FBS. SIRC are grown in MEM supplemented with 10% FBS. Viral infections (moi of 5) were carried out using 17 syn+ EGFP strains of HSV-1 [8]. Receptor tyrosine kinase inhibitor Tyrphostin AG 1007 was purchased from Sigma-Aldrich Co. LLC.(St. Louis, MO, Cat#: T5943). ACV was acquired from Sigma-Aldrich Co. LLC.(St. Louis, MO, Cat#: A4669) and the concentration used for blocking viral replication was 100 μM [9]. Inactivation of virus was performed using a UV crosslinker (Spectroline Select^™^ Series UV Crosslinkers, model#: XLE-1000, 254nm) and the condition is 120mJ/cm^2^.

### 2.1 Western Blotting

The protocol in this study was described previously [2] with minor modification. Protein extract was subjected to gel electrophoresis and transferred using iBlot® Gel Transfer Device (Cat#: IB1001) from Invitrogen ( Carlsbad, CA) essentially described by the manufacturer. Rabbit anti-Egr-1 polyclonal antibody (Santa Cruz SC-110x) was used at a dilution of 1:1,000 for detecting the presence of Egr-1. Anti-α-Tubulin mouse antibody (Calbiochem, Cat#: CP06, San Diego, CA) was added at a dilution of 1:10,000 as control. The chemiluminiscent signal from the membranes was detected by Bio-Rad Chemi-docl XRS imaging systems (Hercules, CA).

### 2.2 Immunofluoroscence

The protocol was described previously[2]. In short, approximately 20,000 cells were placed in a multichamber slide (Cat# 354104, BD Falcon) with respective media a day before infection. Infected cells at 24 hpi were rinsed once with 2 ml PBS for 5 min and fixed with −20°C 100% methanol. Slides were incubated with 2% normal blocking serum followed by incubation with primary antibody for overnight at 4°C. The slides were then incubated with fluorescent conjugated secondary antibody for 1 h at RT. Finally the slides were mounted with fluorescent mounting medium containing DAPI for fluorescent microscopy using Olympus Inverted microscope 1X71 and images were acquired and processed using CellSens Software from Olympus America Inc. (Center Valley, PA).

### 2.3 Recombinant HSV-1 Expressing Egr-1 and GFP

The detailed procedure was essentially explained previously[10]. To sum up, subcloned Egr-1 was introduced into the pEGFP vector between the ClaI and PacI sites by PCR using primers containing these two restriction sites (5′-GCCGTCATCGATGAAGAATCTGCTTAGGGTTAG-3′; and PolyA REV: 5′-CGCCACCCGAGATACCGAAGAAATTAATTGAATGG-3′; restriction cleavage sites underlined). This plasmid pEGFP-Egr1 was cotransfected with pYEbac102 (bacterial artificial chromosome containing complete HSV-1 genome) into 293HEK cells followed by infection of recombinant adenovirus, AxCANCre (adenovirus expressing Cre recombinase) to enhance the CRE recombination via Lox P sites. Media collected at 22 hrs post-transfection were subjected to infection of Vero cells followed by three-rounds of green fluorescent plaque purification. The BAC was inserted into the intergenic region between the U_L_3 and U_L_4 genes during the original construction of HSV-1 pYEbac102 and the viruses generated by this method retained full capacity of replication and pathogenecity [8]. Schematic representation of the construction is described in Fig. 4A. Noted that GFP was driven under the control of the elongation factor 1α (EF-1 α) promoter.

### 2.4 qRT-PCR

The protocol of quantitative RT-PCR (qRT-PCR) was provided by the manufacturer(Bio-Rad, Cat #170-8892). In short, 0.5 μg of total RNA and primer pair of interest was subjected to iScript^™^ One-Step RT-PCR Kit with SYBR^®^ Green (Bio-Rad, Cat #170-8892) and the reaction was performed on Bio-Rad MyiQ Single-color real-time PCR detection system (Cat#: 170-9740). The relative fold of expression of Egr-1 and ICP0 were analyzed by measuring Δ ΔCt normalized by GFP (for ICP0) or cellular genes PPIA and PGK1 (for Egr-1). The sequences of the experimental primers are as follows: Egr-1: 5′-AGA CCA GTT ACC CCA GCC AAA C-3′ and 5′-AAA ATG TCA GTG TTC GGC GTG-3′; GFP: 5′-GCA GAA GAA CGG CAT CAA GGT G-3′ and 5′-TGG GTG CTC AGG TAG TGG TTG TC-3′ ′; ICP0: 5′-TTC GGT CTC CGC CTG AGA GT -3′ and 5′-GAC CCT CCA GCC GCA TAC GA -3′; Peptidylprolyl isomerase A (PPIA): 5′-AGC ATA CGG GTC CTG GCA TCT-3′ and 5′-CAT GCT TGC CAT CCA ACC ACT CA-3′[11]; Phosphoglycerate kinase 1 (PGK1): 5′-ACC TGC TGG CTG GAT GGG CTT-3′ and 5′-GCT TAG CCC GAG TGA CAG CCT C-3′[11]. The reaction was carried out at 50°C for 20 min, 94°C for 2 min, and followed by 35 cycles of 94°C for 30 s, 61°C for 30 s, and 68°C for 30 s.

### 2.5 Quantitative Analysis of Egr-1 Protein Synthesis

This method is based on Qiagen’s Cignal^™^ Reporter Assay (Cat#: CCS-8021L). In short, 20,000 cells were transfected by control plasmid or Egr-1 Reporter plasmid pCignal-ERE containing EGR1-responsive firefly luciferase and a constitutively expressing Renilla luciferase constructs. At 24 h post transfection, the cells were subjected to viral infection at moi of 5 followed by Dual Luciferase Assay (Promega, Cat#: E1910) using GloMax® 20/20 Luminometer (Promega, Cat#: E-5311) at 24 hpi. Negative and positive controls were provided by the manufacturer and included in the experiments. The results were analyzed by a Student’s paired *t* test with a two-tailed distribution (Microsoft Excel).

### 2.6 Chromatin-Immuno Precipitation (ChIP)

The protocol was essentially described previously [2] with modification. In short, cell monolayers were treated with 1% formaldehyde solution for 10 min at room temperature then harvested and subjected to sonication. The lysed samples were centrifuged for 10 min at 13,000 rpm at 4°C, and the supernatant was diluted 10-fold with RIPA buffer containing protease inhibitor. Immunoprecipitation was then performed with Dynabeads Protein A (Invitrogen, Cat#: 100.01D) with pre-immune IgG (Cat#: 2729; Cell Signaling; Boston, MA) as control followed by addition of anti-Egr-1 Ab (Cat#: 4153; Cell Signaling; Boston, MA). To analyze immuno-precipitated DNA, PCR amplification was performed with primers of ICP22: 5′-TGG GGT GGG CGG GTC TTT C-3′ and 5′-ACG AAC GAC GGG AGC GGC TG-3; ICP0: 5′-TAA TGG GGT TCT TTG GGG GAC ACC-3′ and 5′-TGC AAA TGC GAC CAG ACT GTC-3′. The products were analyzed by 2% agarose gel electrophoresis to verify the quality of the PCR primers and by quantitative PCR (qPCR) using Percent-Iuput Method (PIM)to measure the Egr-1 occupancy. In short, the input of PIM represents the amount of chromatin used in the ChIP and the experimental sample signals obtained from the ChIP qPCR were divided by signals obtained from the respective input samples to reflect the true binding of the protein to the target DNA. In this study, 1% of starting chromatin was collected/used as input and a dilution factor of 100 or 6.644 cycles (i.e., log_2_ of 100) is subtracted from the Ct value of diluted input ((Life Technology, CA).

### 2.7 Plaque Assays

Vero monolayers with 100% confluency were incubated with 200 μl of supernatant at different dilutions for 45 min in 24-well plates on a rocking platform followed by addition of 1 ml fresh medium (EMEM containing Earle’s Balanced Salt Solution, non-essential amino acids, 2 mM L-glutamine, 1 mM sodium pyruvate, 1500 mg/L sodium bicarbonate, and 20% FBS) to each well. After 48 hpi, the infected cells were first washed with PBS 2 times then treated with crystal violet (PML Microbiologicals, Wilsonville, OR) for 10 min finished by washing with water. Plaques (in triplicates) were counted in each well and analyzed by a Student’s paired *t* test with a two-tailed distribution (Microsoft Excel). The titer of the recombinant virus was determined by comparing the GFP expression to the wild-type virus using qRTPCR at 10 hpi.

## 3. RESULTS

### 3.1 Viral Binding and Entry to Cells are not Sufficient to Produce Egr-1 Protein

**Fig. 1:**
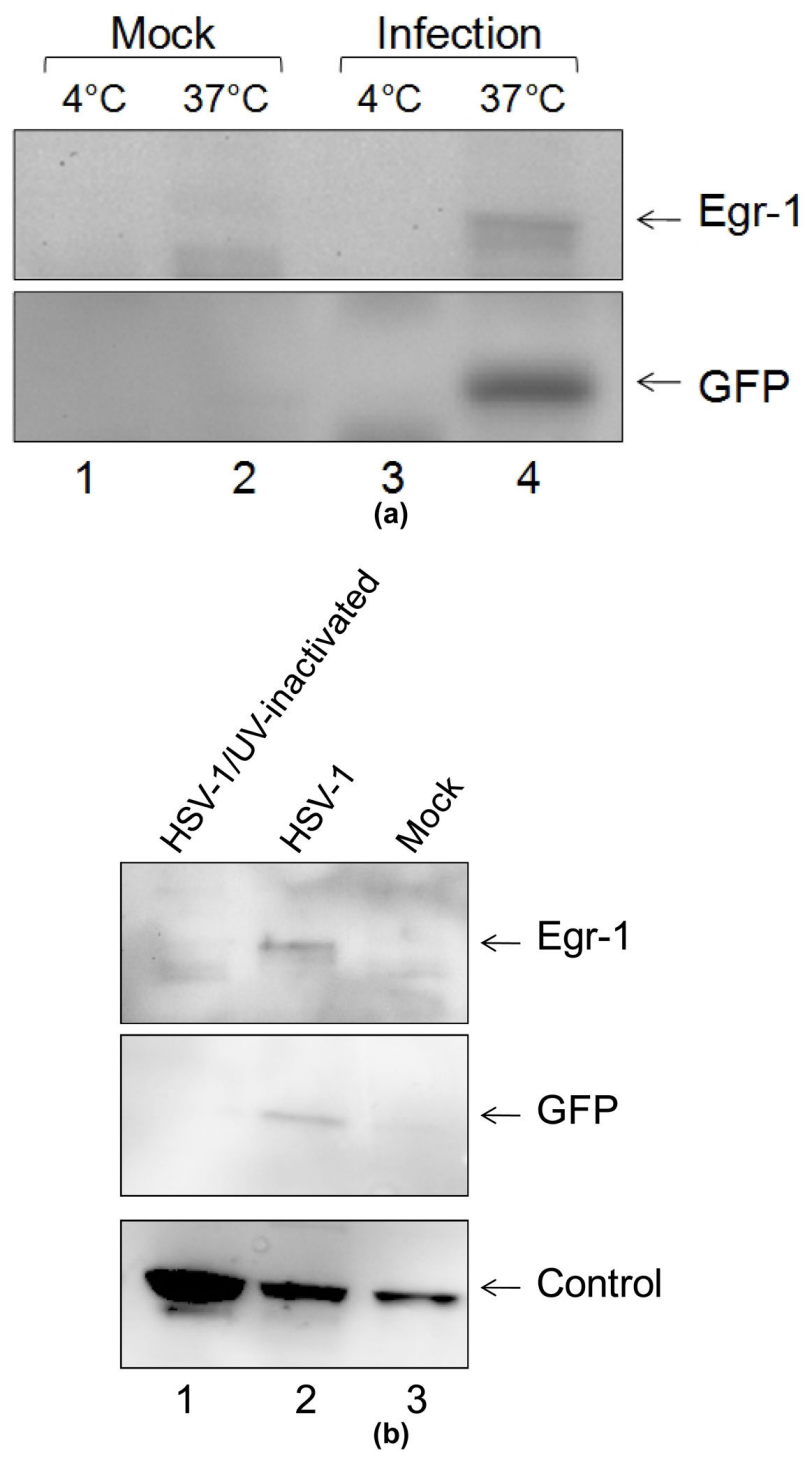

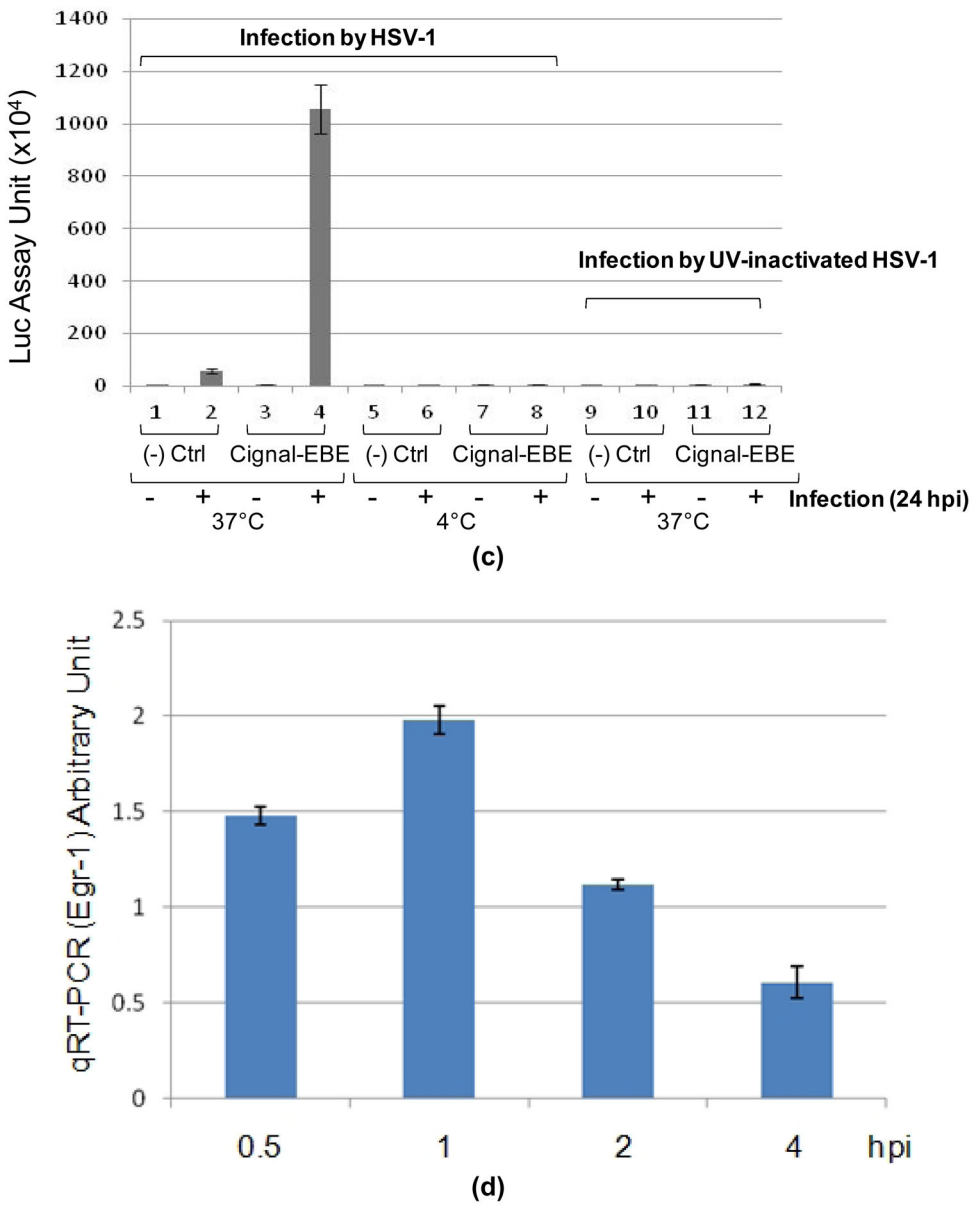
Egr-1 induced by HSV-1 infection required active viral gene expression (a). SIRC cells were infected by 17 syn+ EGFP strains of HSV-1 at 4°C or 37°C and subjected to Western blot analyses at 24 hpi using antibodies against Egr-1, GFP (infection control), and α-Tubulin (loading control).(b). Infection described in a. was performed using regular or UV-inactivated virus at 37°C and the cell lysates were collected at 24 hpi followed by Western blot analyses.(c). Same infection shown in a. and b. were performed analyzed by quantitative Cignal^™^ Reporter Assay.(d). qRTPCR was performed to compare the accumulation of Egr-1 transcript at different time points (0.5, 1, 2, and 4 hpi). The amount of Egr-1 mRNA was normalized by two cellular genes PPIA and PGK1 which were reported not to be affected by HSV-1 infection (materials and methods)

To determine if the virus attachment to cells can trigger the synthesis of Egr-1, we performed infections at 4°C followed by Western blot analysis. The results showed that SIRC cells when infected at 4°C (viruses attached to cell surface without entry) was not sufficient to produce Egr-1 protein at 24 hpi. (Fig. 1A). In addition, infection by UV-inactivated virus, which allows entry but no viral transcription, did not induce Egr-1 (Fig. 1B). These observations were further investigated by Cignal^™^ Reporter Assay and the data confirmed and indicated that infections at 4°C or by UV-inactivated virus exhibited approximately 1,000-fold decrease of Egr-1 induction compared to normal infections at 37°C (Fig. 1C). Additional studies showed that Egr-1 protein was not detected until 24 hpi but the mRNA peaked at 1 hpi and declined afterwards (Fig. 1D). Together these experiments demonstrated that binding and entry of the virus to cells are not enough to induce Egr-1 protein. More episodes from the virus are required for Egr-1 induction.

### 3.2 Viral Replication is not Necessary to Stimulate Egr-1

**Fig. 2:**
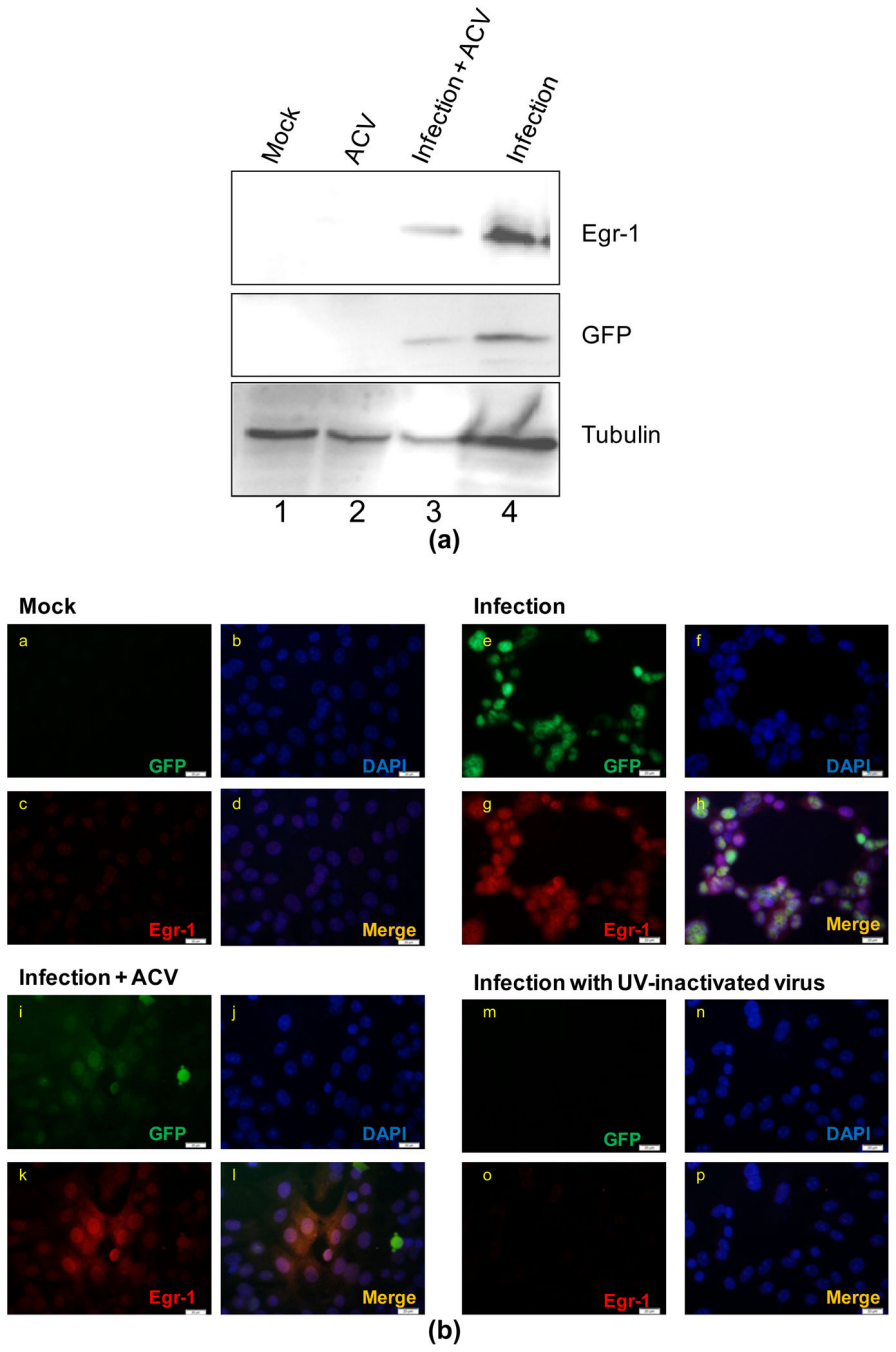
Egr-1 induction does not require viral replication. (a). Infections of SIRC with 17 syn+ EGFP strains of HSV-1 were performed in the presence or absence of ACV followed by Western blot analyses at 24 hpi using antibodies against Egr-1, GFP (infection control), and α-Tubulin. (b). Immunofluorescent microscopy was done at 24 hpi to validate the observation in a. Egr-1 was effectively produced even without the replication (compare c, g, k, and o). Green fluorescent signals represent the cells infected by viruses

Viral DNA replication marks an important feature during the HSV-1 life cycle. Therefore we investigated Egr-1 induction upon viral infection in the absence of viral replication by adding acyclovir (ACV) to block the viral DNA synthesis. The Western blot analyses showed that ACV treatment did not abolish the Egr-1 stimulation (Fig. 2A). Additional study using immunofluorescent microscopy showed that the Egr-1 signals coincided with the viral infection (GFP fluorescence from the recombinant virus), supporting the previous observation that Egr-1 was successfully stimulated after viral entry and transcription prior to the replication (Fig. 2B). It also confirmed the results of Western blot analyses and further demonstrated that viral gene expression is required for efficient Egr-1 induction since UV-inactivated virus failed to stimulate Egr-1 expression (Panel m and o, Fig. 2B).

### 3.3 Binding of Induced Egr-1 to HSV-1 Promoters

**Fig. 3:**
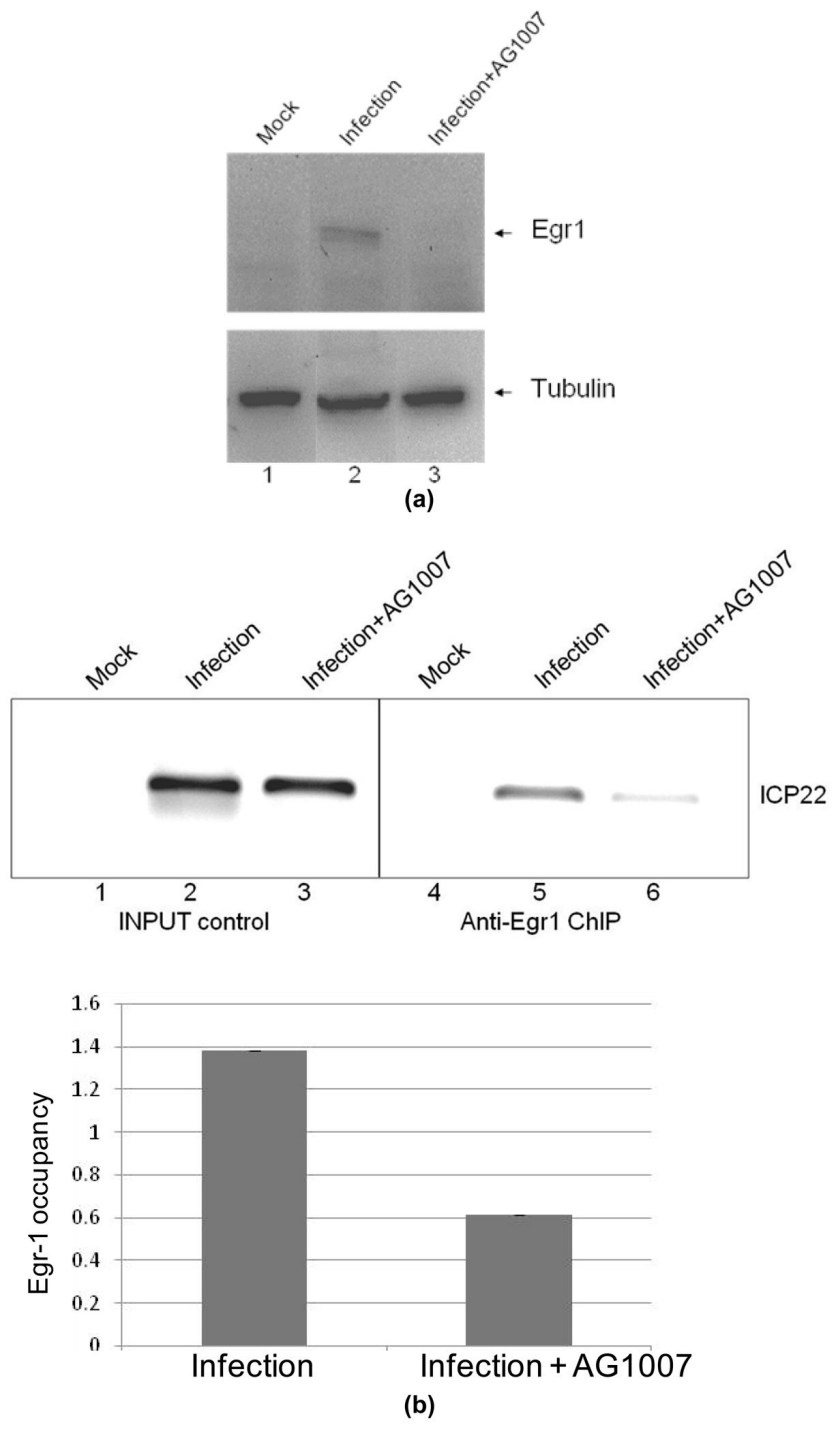

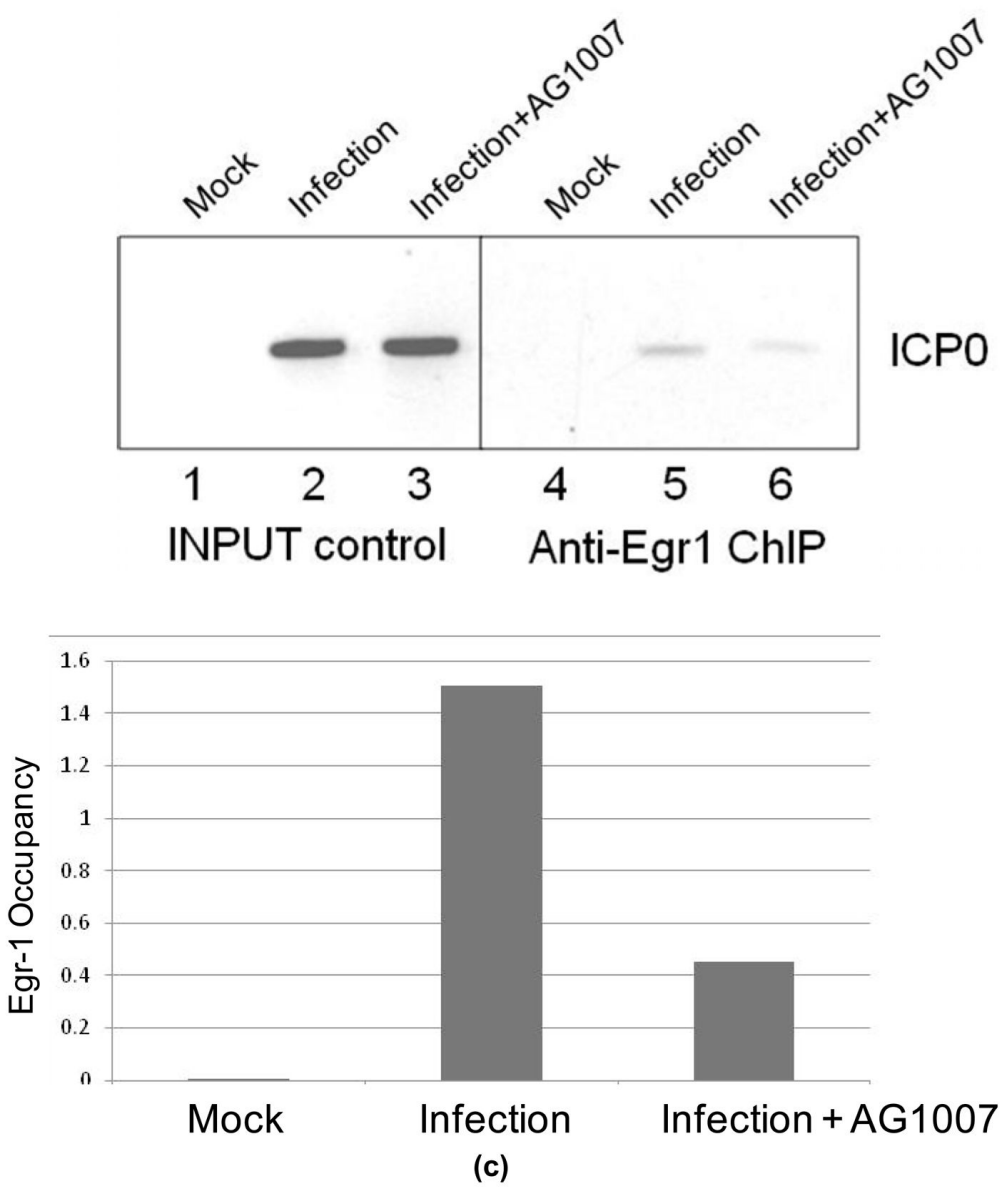
HSV-1 IE regulatory sequences were bound by induced Egr-1. (a). Infections of SIRC cells at moi of 5 with a receptor tyrosine kinase inhibitor AG1007 at 1μM were compared to infection without the inhibitor by Western Blot analyses.(b). Same infections described in 3a were performed with or without AG1007 followed by ChIP via regular and qPCR using ICP22 primers against the EBE to address the Egr-1 occupancy. The binding of Egr-1 to ICP22 EBE was confirmed and the interaction was decreased to 42% by AG1007 treatment. (c). The Egr-1 occupancy on ICP0 promoter was analyzed by ChIP described in 3b. The quantitative PCR indicated that Egr-1 recruitment to ICP0 promoter was reduced to 28% by AG1007

Previously we identified Egr-1 Binding Elements (EBE) within HSV-1 key regulatory sequences and over-expressed Egr-1 was able to bind to not only EBE but GC-boxes of HSV-1 IE promoters using transient transfection studies[2]. Current studies are focusing on if induced Egr-1 by infection can be recruited to HSV-1 promoters during infection. Prior to the investigation, a number of signaling inhibitors were tested to determine their putative inhibitory effects on Egr-1 expression. The results indicated that a receptor tyrosine kinase inhibitor AG1007 at 1μM exhibited potent inhibition on Egr-1 upon infection (Fig. 3A). The exact mechanisms are under investigation.

ChIP was performed to study the occupancy of induced Egr-1 on viral regulatory sequences. The results revealed that induced Egr-1 was able to bind to ICP22 EBE (Fig. 3B) as well as ICP0 GC-box which is an alternative Egr-1 binding element (Fig. 3C). AG1007 treatment decreased the signals, suggesting that Egr-1 interaction to ICP22 and ICP0 regulatory sequences were diminished due to reduced Egr-1 stimulation (Fig. 3B and C). Collectively these results demonstrated that Egr-1 induced by infection may be recruited to HSV-1 key sequences and potentially has regulatory effects on viral gene expression and replication.

### 3.4 Egr-1 Overexpression Enhanced HSV-1 Gene Expression

**Fig. 4:**
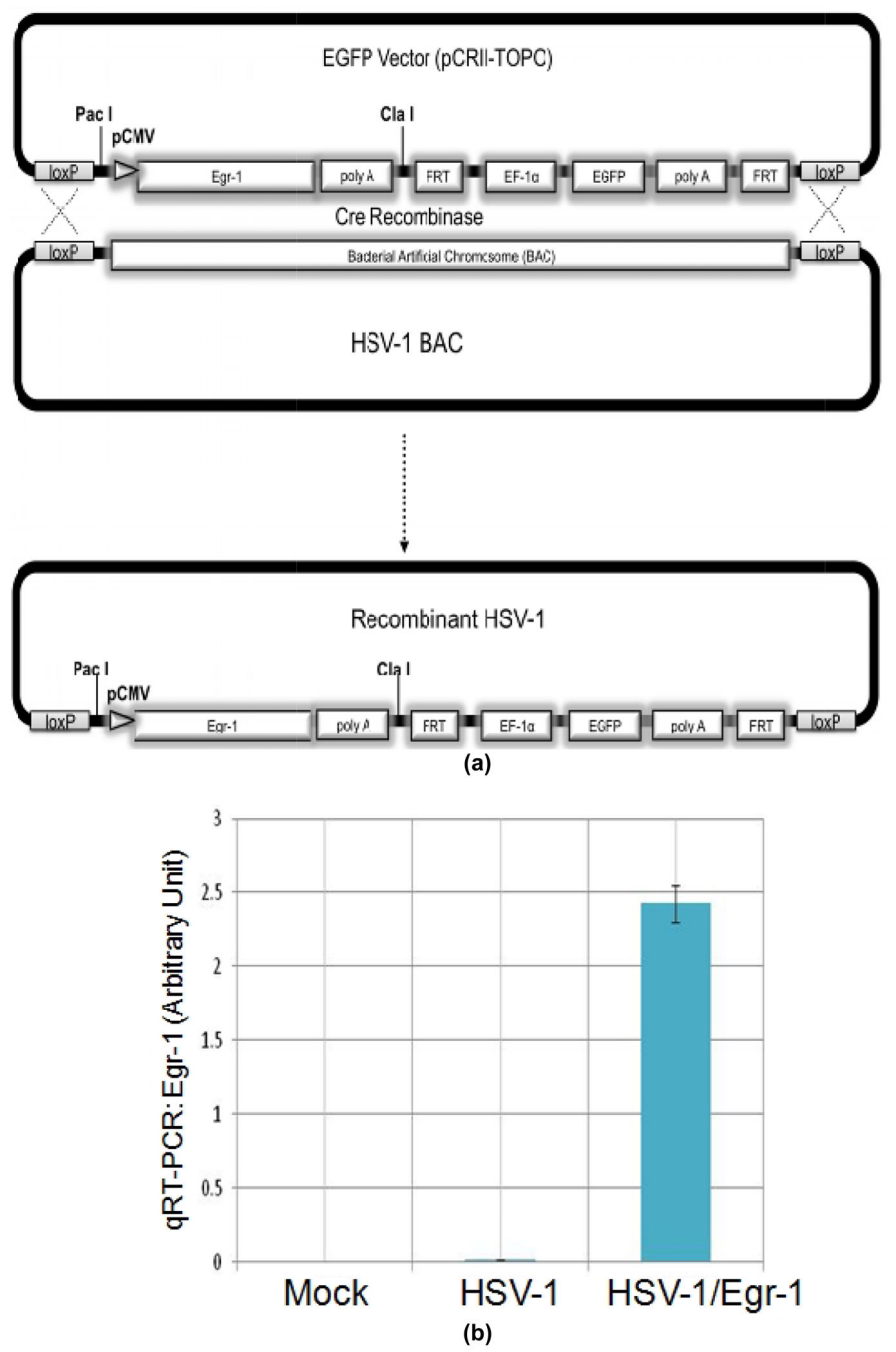

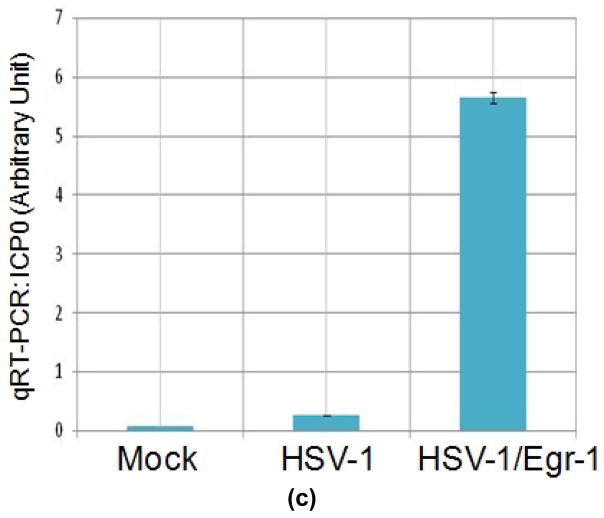
Construction of recombinant virus over-expressing Egr-1 and its regulatory effects on HSV-1.(a). Schematic representation of recombinant virus construction. (b). Infection of SIRC cells followed by RNA isolation and qRT-PCR using primers against open reading frames of Egr-1 and ICP0.(c). The same experiments described in 4b were performed to analyze ICP0 expression profile

Recombinant HSV-1 over-expressing Egr-1 (HSV-1/Egr-1) was generated based on the method described previously (Fig. 4A) and the titer was determined by comparing the GFP expression to the wild-type virus using qRTPCR at 10 hpi (data not shown). Infection of SIRC cells indicated that recombinant virus HSV-1/Egr-1 produced a 121-fold increase of Egr-1 compared to the original wild-type virus at 10 hpi (Fig. 4B). Additional studies further showed that ICP0 expression was prolonged and increased at 10 hpi by as many as 22.6-fold (Fig. 4C). Unpublished data revealed that ICP22 was also enhanced (not shown). These results demonstrated that Egr-1 over-expression increased the expression of HSV-1 IE genes and may have implication on the viral replication and life cycle.

### 3.5 Egr-1 Promoted HSV-1 Replication and Release of Infectious Viruses

**Fig. 5:**
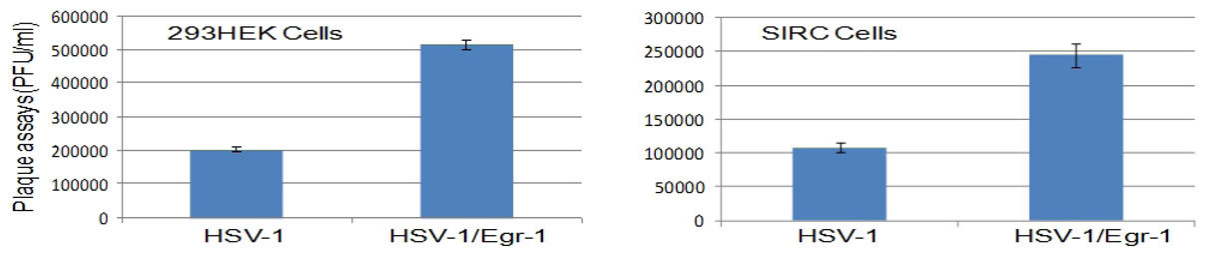
Egr-1 promoted HSV-1 replication and release of infectious viruses. The plaque assays were performed by infecting Vero cells using supernatant from infections of 293HEK and SIRC cells with wild-type and recombinant viruses

The effects of Egr-1 on HSV-1 replication were tested by plaque assays using wild-type and recombinant viruses. The experiment was first performed on 293 cells in which the Egr-1 was not induced by viral infection[2]. The results showed that Egr-1 over-expression exhibited a 2.5-fold increase of infectious virus release (Fig. 5, left panel). Similar data was observed when the SIRC cells were used for plaque assays. Recombinant virus expressing Egr-1 increased the release of infectious viruses by approximately 2.5-fold (Fig. 5, right panel). Furthermore, co-transfection of HSV-1/Bac (containing complete HSV-1 genome) and Egr-1 over-expression vector in SIRC cells also exhibited increased replication via plaque assays (data not shown). These assays collectively demonstrated that the prolonged/over-expression of Egr-1 enhanced the viral replication, presumably via increased immediate-early (IE) gene expression.

## 4. DISCUSSION

Egr-1 influences a variety of divergent cellular functions such as survival, apoptosis, growth, growth arrest, differentiation, transformation, etc[12]. We have previously demonstrated that Egr-1 was induced upon HSV-1 infection in epithelium and corneal cells and this observation implied that Egr-1 may participate in host cellular responses to modulate HSV-1 infections [2]. Subsequent studies indicated that Egr-1 was produced after HSV-1 infection in cornea and inhibition of production decreased the virus-mediated pathogenesis[13]. However, the mechanisms are not clear.

We showed that Egr-1 transcript can be detected an hour after infection (2). This rapid induction prompted us to hypothesize the binding of the virus to target cells may trigger the protein synthesis. However, our results did not support the hypothesis since the infection at 4°C in which viruses attached without entry showed no evidence of Egr-1 induction (Fig. 1A and 1C), suggesting that episodes after entry is required for successful protein expression. Additional experiments using UV-inactivated viruses indicated that *de novo* viral protein synthesis is necessary for this unique induction. To investigate the contribution of viral replication in this induction, ACV was used to block the HSV-1 DNA synthesis and late viral gene expression. The results revealed clear sign of Egr-1 expression (Fig. 2A and 2B). Together, these observations strongly suggest that Egr-1 induction requires HSV-1 protein expression prior to the viral DNA replication and tegument proteins, if involved, are not sufficient. Nevertheless, our preliminary data revealed partial Egr-1 transcript was detected upon viral infection at 4°C or by UV-inactivated viruses (data not shown). The mechanisms are not understood but it is likely that viral proteins facilitated the Egr-1 translation or prevented premature transcription termination. More studies are underway to investigate this novel phenomenon.

Our attempts to block Egr-1 expression using a number of inhibitors against signaling pathways revealed one hit, a receptor tyrosine kinase inhibitor Tyrphostin AG 1007, which completely blocked the expression of Egr-1, evidenced by Western blot analyses and immune-fluorescent microscopy (Fig. 3A and 3B). Using ChIP with or without AG1007, we demonstrated that HSV-1 induced Egr-1 was sufficient to bind to HSV-1 IE promoters such as ICP22 (Fig. 3C) and ICP0, presumably to the Sp1 since Egr-1 was shown to occupy these sites[14]. It is likely that induced Egr-1 may generate novel regulatory effects on HSV-1 gene expression and pathogenic consequences through direct binding on the viral genome or by modulating host responses. Additional experiments using recombinant virus implied that Egr-1 over-expression increased the release of infectious viruses and replication probably by promoting IE gene expression such as ICP0, etc. The mechanisms of increasing ICP0 and ICP22 expression by Egr-1 are unclear. It is perhaps due to the recruitment of p300 to these IE promoters by Egr-1 [15–17]. The Egr-1/p300 complex may promote transcription by loosening the chromatin structure via its intrinsic histone acetyltransferase activity.

The effects of Egr-1 on HSV-1 gene expression during acute and latent infections are controversial. Induction of the EGR transcription factor family in the nervous system has been linked to stress and stimuli[18]and the same conditions are known to initiates HSV-1 reactivation [19]. However, the roles of EGR in the regulation HSV-1 biology were not extensively studied. Early investigation identified an EGR consensus sequence immediately downstream of the TATA box of LAT promoter[20]. Subsequent cotransfection analyses showed that Egr-2 but not Egr-1 and Egr-3 reduced the LAT promoter activity. This observation proposed a connection between neuronal stress and HSV reactivation from latency, suggesting that activation of the Egr-2 from the same stress/stimuli responses may result in LAT suppression and thus play a role in reactivation by abolishing latency. Additional and current studies indicated that during acute infection HSV-1 can directly induce Egr-1 and enhance viral gene expression and replication [2,13,21]. All these results supported the roles of EGR proteins in suppressing latency and promote lytic infection. Nevertheless, Egr-1 expression was first detected and characterized while treating PC-12 cells[3]and dorsal root ganglion (DRG) neurons[22]with nerve growth factor (NGF). NGF has been shown by several laboratories to mediate HSV-1 quiescent infection in various systems such as peripheral sympathetic and sensory neurons in vitro[23,24]and in PC-12 cells[25–27]. Given the fact that PC-12 cells and DRG neurons under NGF influence are frequently used as models for HSV-1 latency study, it would suggest that NGF-mediated HSV-1 gene silencing and quiescent state may result from, at least in part, by Egr-1 triggered inhibition on viral gene expression. This hypothesis was further supported by observation that over-expression of Egr-1 was sufficient to repress HSV-1 ICP4 and ICP22 in chromatin context in vitro[14]. We therefore hypothesized that Egr-1 may exhibit positive or negative regulatory effects on HSV-1 gene expression and replication depending upon distinct specific conditions and cellular environment. At least six different scenarios may explain the diversity. 1. Choice of EGR binding sites: EGR Binding Elements (EBE) were characterized[4]and additional studies indicated that EGR proteins can compete and occupy GC-rich sequences such as Sp1 sites. Previously we identified a novel EBE located in HSV-1 ICP22 intron repeated five times and both ICP22 and ICP4 were controlled during Egr-1 over-expression [14]. It is likely that the amount of Egr-1 may determine the destination of protein binding and the differential interaction may decide the fate of regulation. 2. Timing and duration of Egr-1: Current study showed that viral gene products were required to induce the Egr-1 and various amount of viruses may produce different quantity of Egr-1 within the infected cells. It is shown that the kinetics of Egr-1 induction vary in corneal cells and neurons during HSV-1 infection. Induction of Egr-1 in corneal/epithelial cells was rapid and efficient (unpublished data). The transcript of Egr-1 reached its peak at 1 hour post-infection (hpi) and quickly declined afterwards (personal observation). On the other hand in neuronal cell line, the Egr-1 mRNA was not observed until 12 hpi and the climax of expression occurred after 48 hpi[21]. The significant difference of expression profile may provide clues regarding the roles of Egr-1 in HSV-1 regulation. 3. Presence/availability of HSV-1 tegument proteins: HSV-1 infects a number of different cells using diverse routes. This phenomenon could modulate the Egr-1 function via the presence of viral tegument proteins such as VP16, etc. Egr-1 regulatory effects on HSV-1 IE genes may be altered by the presence of VP16 through protein-protein interaction, discrepancy of EBE targeting, coactivator/corepressor recruitment, etc. In addition, important host factors interacted with VP16 such as OCT-1 and HCF-1 were either not expressed in neurons of sensory ganglia [28] or not localized in nuclei of sensory neurons[29]. Therefore Egr-1 may switch its regulatory effects without VP16/HCF-1/OCT-1 complex in nuclei of neurons. 4. Nucleosomal structure of viral genome: Egr-1 contains domains responsible for activation or repression [30,31], and the decision of cofactor recruiting may be decided by nucleosomal structure on the viral DNA or *vice versa*, particularly in latently infected neurons. For example, Nab2 (NGFI-A-binding protein 2 or Egr-1-binding protein 2) was a nuclear protein found to inhibit transcription induced by EGR particularly in chromatin context via nucleosomal remodeling [32]. This hypothesis was encouraged and supported by a previous study using a stably transformed nucleosome-associated plasmids in which HSV-1 ICP4 and ICP22 were repressed by Egr-1 and Nab2 was recruited to the regulatory sequence of ICP22 intron[14]. 5. Cellular environment under stress and stimuli: host cell signaling pathways responded while the stress and/or stimuli were detected. Egr-1 and other cellular factors may be produced accordingly at different stages of HSV-1 latency (initiation, establishment, maintenance, and reactivation) in neurons and therefore generate divergent regulatory effects. 6. Disparity of cell condition under differentiation: HSV-1 latency was generally believed to occur in differentiated neurons and the expression profiles of host cellular factors can be modified quickly under physiological influence. For example, it is hypothesized that hormonal imbalance may change the latency status through differential EGR isoform expression. The occupancy competition of various EBE by distinct EGR may explain the conflicting regulation during HSV-1 infection.

## 5. CONCLUSION

These results concluded that HSV-1 rapidly triggered the expression of an important host protein Egr-1 and it required viral gene products. The induced Egr-1 bound to viral regulatory elements to regulate the viral gene expression and replication.

## ABBREVIATION

Egr-1: Early Growth Response Protein 1
HSV-1: Herpes Simplex Virus Type 1
IE: Immediate-early Gene
ChIP: Chromatin immune-precipitation
moi: Multiplicity of infection
hpi: Hours post infection
ACV: Acyclovir
